# Influenza and hepatitis B vaccination coverage among healthcare workers in Croatian hospitals: a series of cross-sectional surveys, 2006–2011

**DOI:** 10.1186/1471-2334-13-520

**Published:** 2013-11-05

**Authors:** Rok Civljak, Neven Papic, Valerija Stamenic, Smilja Kalenic, Ilija Kuzman, Josip Car

**Affiliations:** 1 Dr. Fran Mihaljevic University Hospital for Infectious Diseases, Mirogojska 8, 10000 Zagreb, Croatia; 2 Department of Infectious Diseases, Medical School, University of Zagreb, Šalata 3, 10 000 Zagreb, Croatia; 3 Department for Projects and Programs, Directorate for Medical Affairs, Ministry of Health, Ksaver 200a, 10 000 Zagreb, Croatia; 4 Department of Bacteriology, Virology and Parasitology, Medical School, University of Zagreb, Šalata 3, 10000 Zagreb, Croatia; 5 School of Public Health, Imperial College London, Reynolds building, London, UK

**Keywords:** Influenza, Hepatitis B, Healthcare workers, Vaccination

## Abstract

**Background:**

Healthcare workers (HCWs) are at an increased risk of exposure to and transmission of infectious diseases. Vaccination lowers morbidity and mortality of HCWs and their patients. To assess vaccination coverage for influenza and hepatitis B virus (HBV) among HCWs in Croatian hospitals, we conducted yearly nationwide surveys.

**Methods:**

From 2006 to 2011, all 66 Croatian public hospitals, representing 43–60% of all the HCWs in Croatia, were included. Statistical analysis was performed using the Kruskal–Wallis analysis of variance, Dunn’s multiple comparison analysis and the chi-square test, as appropriate.

**Results:**

The median seasonal influenza vaccination coverage rates in pre-pandemic (2006–2008) seasons were 36%, 25% and 29%, respectively. By occupation, influenza vaccination rates among physicians were 33 ± 21%, 33 ± 22% among graduate nurses, 30±34% among other HCWs, 26 ± 21% among housekeeping and the lowest, 23 ± 17%, among practical nurses (p < 0.01). In 2009–2010 season, seasonal influenza vaccination coverage was 30%, while overall vaccination coverage against pandemic influenza was fewer than 5%. Median vaccination coverage in the post-pandemic seasons of 2010–2011 and 2011–2012 decreased to 15% and 14%, respectively (reduction of 24% and 35%, respectively, p < 0.0001). Meanwhile, the median mandatory HBV vaccination coverage was 98%, albeit with considerable differences according to work setting (range 19–100%) and occupation (range 4–100%).

**Conclusions:**

We found substantial year-on-year variations in seasonal influenza vaccination rates, with reduction in post pandemic influenza seasons. HBV vaccination is satisfactory compared to seasonal influenza vaccination coverage, although substantial variations by occupation and work setting were observed. These findings highlight the need for national strategies that optimize vaccination coverage among HCWs in Croatian hospitals. Further studies are needed to establish the potential role of mandatory vaccination for seasonal influenza.

## Background

Healthcare workers (HCWs), due to direct and indirect contact with patients, are at an increased risk of exposure to and transmission of infectious diseases [1-5]. In Croatia, the majority of vaccine-preventable infectious diseases, such as diphtheria, tetanus, pertussis, poliomyelitis, measles, mumps, rubella and tuberculosis, are covered by the national mandatory immunization program for children [6-8].

Vaccination of HCWs against hepatitis B virus (HBV) began to be introduced in Croatia in the 1990s and for many years has been mandatory and free of charge [9,10]. It is performed using a vaccine obtained from a surface antigen of the hepatitis B virus through genetic engineering that is administered in three doses according to a scheme of 0, 1 and 6 months. The immunization of persons who have been exposed to contaminated material is performed by injecting four doses of vaccine according to a scheme of 0, 1, 2 and 12 months.

The annual plan of immunization against infectious diseases is conducted according to the immunization program, which is adopted by the Minister of health at the proposal of the Department of Infectious Disease Epidemiology of the Croatian National Institute of Public Health (CNIPH). The vaccine is provided by the CNIPH to the epidemiology departments, including hospital settings. All HCWs, including medical/nursing students and all new employees, are covered, so all HCWs are supposed to be vaccinated at least by the time they begin their professional careers [9,10]. An ordinance on the prevention and control of hospital infections from 2002 places special emphasis on the education and protection of new medical professionals, which has resulted in stricter enforcement measures, especially among newly recruited employees who cannot be hired until they have been vaccinated against hepatitis B. HBV vaccination and post-exposure management after occupational exposure became integral components of a comprehensive program to prevent infections following bloodborne pathogen exposure and important elements of workplace safety [2,3,11,12]. Our study represents the first assessment of this program.

On the other hand, the first official recommendations for influenza vaccination and free immunization programs for HCWs have been in existence since 1984, when the Advisory Committee on Immunization Practices in the USA recommended annual influenza vaccination as the first and best protection against influenza [13]. However, the vaccination of HCWs against influenza is indicated not only for the personal protection of the vaccinated HCWs but also because it contributes to the prevention of influenza among unvaccinated persons in their environment, including their patients and family members [14-17]. A number of studies demonstrated that influenza vaccination of HCWs lowers morbidity and mortality in their patients [15-18].

Despite long-standing recommendations, overall vaccination rates for HCWs in many countries remain unacceptably low, near 40% [5,19-21]. The gap is magnified when one considers the estimate that influenza immunization rates of 80% or higher are essential for providing the herd immunity necessary to reduce healthcare-associated influenza infections substantially, which is generally not the case where vaccination is voluntary [22].

In an effort to combat the low rates of vaccination among HCWs, a growing number of professional medical organizations and healthcare facilities are adopting policies mandating influenza vaccination for individuals who work with patients [23]. This decision is justified by the fact that maximum protection of patients can only be achieved with a high rate of HCWs vaccination [24]. This recommendation is reflected in a 2009 European Union recommendation that set a goal of 75% coverage for this population by 2015 [25]. Mandatory vaccination has been implemented in many countries, thereby demonstrating that an opt-out strategy for influenza immunization significantly improved vaccination rates compared to an opt-in approach and influenza vaccination rates of more than 95% were sustained [26-29].

The CNIPH recommends seasonal influenza vaccination for particularly vulnerable population groups, including HCWs. According to the mandatory immunization program, seroprophylaxis and chemoprophylaxis for specific population groups and individuals at risk are recommended every year prior to the beginning of the flu season (in the autumn), one dose of vaccine that corresponds in composition with the recommendations of the World Health Organization. Vaccination is available free of charge but is not mandatory [7,30].

Using the national surveillance program, we assessed the rates of HBV and influenza vaccination coverage among HCWs in Croatian hospitals. Our study will provide information for the development of a national strategy, including whether influenza vaccination should be mandatory, as is HBV vaccination.

## Methods

### Study design and settings

As part of the ongoing national surveillance program, the National Hospital Infection Control Advisory Committee (NHICAC) conducted a series of nationwide cross-sectional surveys from 2006 to 2011 among HCWs in Croatian hospitals.

### Data collection

We distributed questionnaires to all Croatian public hospitals: 36 acute care (14 university and 22 general) and 30 long-term care and specialized hospitals, with a total of over 30,000 employees (ranging from 29,182 to 35,343 HCWs annually), representing 43–60% of all HCWs in the Croatian healthcare system. The survey was conducted by the members of the Hospital Infection Control Advisory Committee of each hospital. Each hospital was required to provide annual vaccination rates for HCWs, both overall and by occupation. In our analysis, HCWs were stratified into physicians (including residents), nurses (practical nurses with high school qualifications and graduate nurses with three-year university-level nursing degrees), other HCWs (including laboratory, radiology and physical medicine personnel) and housekeeping personnel (as defined by the CDC [31]). Vaccination coverage for agency staff and those staff members who receive their vaccines off-site was also documented. Hospital administrative staff and HCWs from primary care and outpatient healthcare facilities were not included.

### Statistical analysis

Medians with the interquartile ranges of the sets of the reported vaccination rates are presented. The numbers of missing values varied from query to query, which were excluded from the following statistical analysis. Statistical analysis involved a year-by-year comparison using the Kruskal–Wallis analysis of variance, Dunn’s multiple comparison analysis and the chi-square test, as appropriate, using Prism 6 software (GraphPad Software, San Diego, CA, USA); *p* < 0.05 was considered significant.

### Ethics

All data were collected with the consent of hospital administration, as routine data assembled for the National Annual Surveillance Report of the NHICAC of the Croatian Ministry of Health. The NHICAC was prohibited from revealing the identities of individual hospitals in the reports. Therefore, data from individual hospitals were screened using backup codes instead of names or collectively according to hospital groups.

## Results

**Figure 1:**
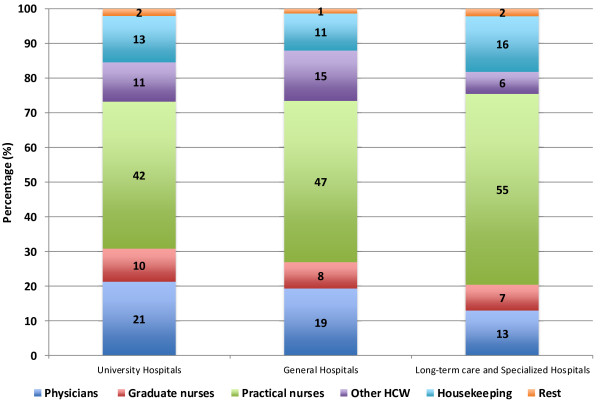
Proportions of healthcare workers included in the study, overall and stratified by type of hospital and occupation, based on data from the national surveillance program of the National Hospital Infection Control Advisory Committee, all 66 public hospitals in Croatia, 2006–2011.

Among the 66 hospitals contacted, the response rate varied between 57% and 83%, depending on the year. Only 33 (50%) of the hospitals provided data on the coverage of various HCW subgroups. The data were provided as percentages or numbers of vaccinated HCWs. The baseline characteristics of the HCWs included in the study, overall and stratified by type of hospital, are presented in Table 1. The structure of the HCWs by occupation did not significantly change during the period studied, so the data for 2010 are shown (Figure 1).

**Table 1:** Healthcare workers included in the study: overall, by type of hospital, by survey year

	Number of HCWs^*^				**% of all HCWs in Croatia**^**§**^
	**Acute care hospitals**		**Long-term care and specialized**	**Total**^**†**^	
**Year**	**University**	**General**			
2006	16.645	10.795	2.690	30.130	43%
2007	14.585	11.879	2.718	29.182	44%
2008	19.019	12.916	2.949	34.884	53%
2009	19.151	13.225	2.967	35.343	54%
2010	18.117	13.853	3.001	34.971	52%
2011	21.870	14.249	3.961	40.080	60%

*HCWs—healthcare workers.

^†^the total number of HCWs in Croatian hospitals that entered the study.

^§^the percentages of respondents in relation to the total number of HCWs employed in Croatia as reported in [6].

### Pre-pandemic seasonal influenza vaccination coverage

**Figure 2:**
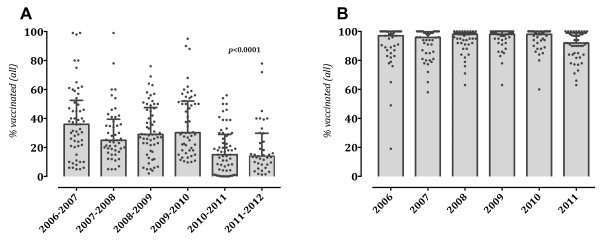
**Seasonal influenza (Panel A) and HBV (Panel B) vaccination coverage among HCWs in Croatian hospitals, stratified by season/year.** Each dot represents one hospital while bars represent the medians with IQRs. Statistical significance was calculated using the Kruskal–Wallis and post hoc multiple comparison tests. In **(Panel A)**, significant decreases in 2010–2011 and 2011–2012 post-pandemic vaccination coverage is seen in comparison to the four pre-pandemic seasons. Meanwhile, HBV vaccination coverage was stable during the period studied (*p* = 0.07) **(Panel B)**.

**Figure 3:**
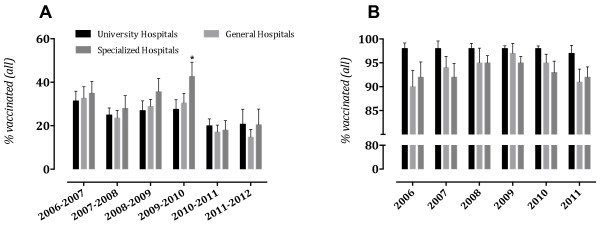
**Seasonal influenza (Panel A) and HBV (Panel B) vaccination coverage among HCWs in Croatian hospitals, stratified by type of hospital.** Medians with interquartile ranges were plotted. In 2009–2010, significant increase in vaccination coverage in long-term care and specialized hospitals was observed (*p* = 0.03) **(Panel A)**. In the following post-pandemic seasons, significant decrease in all the groups was observed (p < 0.0001, Kruskal–Wallis) **(Panel A)**. **(Panel B)** shows significant differences between university and general (*p* = 0.015), and university and long-term care and specialized hospitals (*p* = 0.006).

**Figure 4:**
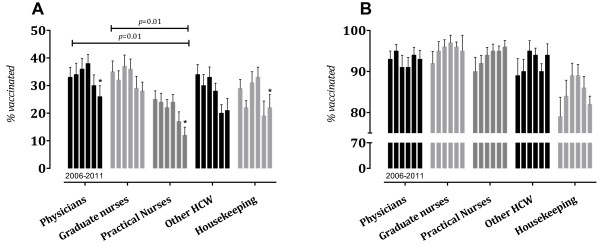
**Seasonal influenza (Panel A) and HBV (Panel B) vaccination coverage among healthcare workers in Croatian hospitals, stratified by occupation and year.** The bars represent medians with IQRs. Statistical significance was calculated using the Kruskal–Wallis and post hoc multiple comparison tests. Statistical analysis showed significant differences among physicians, graduate nurses and practical nurses (p = 0.01). Furthermore, significant decreases in 2011–2012 were observed (*, p < 0.05)) **(Panel A)**. Meanwhile, HBV vaccination coverage was the lowest among housekeeping personnel (p = 0.001) **(Panel B)**.

From the 2006–2007 influenza seasons, the median seasonal influenza vaccination rates at the hospitals studied were 36% (IQR 21–52.5), 25% (IQR 17.5–39.5) and 29% (IQR 20.6–47.5), respectively (Figure 2, Panel A). There were some differences in vaccination coverage between acute care and long-term care facilities, which were not statistically significant (Figure 3, Panel A). By occupation, influenza vaccination was the most common among physicians (30% [IQR 12.7–50.7], 34% [IQR 20–47] and 36% [IQR 20.3–49], respectively), followed by graduate nurses (35% [IQR 14–48], 32% [IQR 16–45.7] and 37% [IQR 19.2–49.5], respectively), other HCWs (34% [IQR 11.3–52.9], 30% [IQR 3–51] and 33% [IQR 12.2–48.2], respectively) and lowest among practical nurses (25% [IQR 13–36], 24% [IQR 12–31.6] and 22% [IQR 10–33.2], *p* < 0.01), and housekeeping staff (29% [IQR 11.7–42.4], 22% [IQR 11.2–30.2] and 21% [IQR 15.2–48.2], respectively). Significant differences among practical nurses, physicians and graduate nurses were observed (Figure 4, Panel A). Further analysis by occupation and type of hospital showed no statistical differences.

### Seasonal and pandemic influenza vaccination coverage in 2009–2010

In 2009, seasonal influenza vaccination in Croatia started in November and showed similar trends as in previous seasons. In total, 7,972 of the HCWs were vaccinated, with a median of 30% (IQR 18–52) of the hospitals studied (Table 2, Figure 2, Panel A). Higher rates in long-term care and specialized hospitals as compared to acute care hospitals (medians of 50% [IQR 25–61] vs. 26% [IQR 17–43] and 23% [IQR 15–35], respectively, *p* = 0.03), were observed (Figure 3, Panel A; Table 2). By occupation, there were no statistical differences, although the rate among physicians was slightly higher than in pre-pandemic seasons (38% [IQR 33–60] in 2009 vs. 30% in 2006) (Figure 4, Panel A).

**Table 2:** Seasonal influenza vaccination coverage among healthcare workers: overall, by type of hospital, three consecutive seasons

	**2009–2010**		**2010–2011**			**2011–2012**		
	**n**	Median (IQR)*	**n**	Median (IQR)*	**% change**^**†**^	**n**	Median (IQR)*	**% change**^**†**^
	**(total)**		**(total)**			**(total)**		
**University hospitals**	4.080	23	2.623	16	–**21%**^**‡**^	2.866	17	**–14%**^**‡**^
	(18.971)	(15–35)	(15.423)	(13–22)		(15.432)	(13–24)	
**General hospitals**	2.847	26	2.340	15	**–23%**^**‡**^	1.376	13	**–53%**^**‡**^
	(9.719)	(17–43)	(10.435)	(7–28)		(9.924)	(3–20)	
**Long-term care and specialized hospitals**	1.045	50	566	13	**–46%**^**‡**^	446	12	**–60%**^**‡**^
	(2.739)	(25–61)	(2.772)	(1–32)		(2.941)	(8–36)	
**Overall**	7.972	30	5.529	15	**–24%**^**‡**^	4686	14	**–35%**^**‡**^
	(31.429)	(18–52)	(28.630)	(4–29)		(28.297)	(8–29)	

*IQR, interquartile range.

^†^decrease in the calculated total number of vaccinated HCWs, as compared to 2009.

^‡^*p* < 0.0001, as measured by the chi-square two-tailed test.

Pandemic influenza vaccination started in the first week of December during the peak of the pandemic in Croatia. Overall, fewer than 1,000 HCWs were vaccinated, i.e., fewer than 5% of all the HCWs in Croatia.

### Post-pandemic seasonal influenza vaccination coverage

The overall vaccination coverage rates in the post-pandemic seasons of 2010–2011 and 2011–2012 were 15% (IQR 4–29) and 14% (IQR 8–29), respectively, which represent a significant decrease by 24% and 35% from 2009 (*p* < 0.0001), respectively (Table 2; Figure 2, Panel A). In the post-pandemic seasons, long-term care and specialized hospitals reported the lowest vaccination coverage (13% [IQR 1–32] and 12% [IQR 8–36], respectively, *p* < 0.0001) (Figure 3, Panel A). Vaccination coverage remained highest among HCWs at university hospitals (16% [IQR 13–22 and 17% [IQR 13–24] in 2010–2011 and 2011–2012, respectively). By occupation, influenza vaccination was the most common among physicians (30% [IQR 20–48] and 26% [IQR 10–34], respectively), followed by graduate nurses (29% [IQR 14–47.5] and 28% [IQR 9.6–44.2], respectively), other HCWs (20% [IQR 8–36] and 21% [IQR 4–48], respectively), housekeeping (19% [IQR 7–32] and 22% [IQR 5–26], respectively) and lowest among practical nurses (17% [IQR 8–29] and 12% [IQR 6–20], respectively, *p* = 0.002). By occupation and work setting, influenza vaccination was most common among physicians who worked in university hospitals (52%, [IQR 37.2–68.5]) and lowest among other HCWs who worked in general hospitals (17%, [IQR 3–28]).

### Hepatitis B vaccination coverage

Meanwhile, from 2006 to 2011, the median HBV vaccination coverage was 98%, albeit with considerable differences according to work setting (range 19–100%) and occupation (range 4–100%) (Figures 2, 3, 4, Panel B). By work settings, HCWs in university hospitals were more likely to receive HBV vaccine than in general or long-term care hospitals (medians 97–98% vs. 87–96% vs. 92–98%, *p* = 0.03, respectively) (Figure 3, Panel B). By occupation, HBV vaccination coverage among physicians was 90–95%, graduate nurses 92–97%, practical nurses 90–96%, other HCWs 89–94% and housekeeping personnel had the lowest vaccination rates (medians 79–89%, *p* = 0.001) (Figure 4, Panel B).

## Discussion

This study presents data for 2006–2011 from the national surveillance program conducted by the NHICAC, which shows that the seasonal influenza vaccination coverage in the pre-pandemic period among HCWs in Croatian hospitals was >30%, with a significant decrease in post-pandemic vaccination coverage from 2006 to 2011 (from 36% to 14%). During the same period in the same population of HCWs, mandatory overall HBV vaccination coverage remained 98%.

The fact that fewer hospitals in our study are reporting data on vaccination coverage against influenza than against HBV reflects different attitudes toward the importance of immunization against these two vaccine-preventable diseases.

Although the average HBV vaccination rate is high, considerable differences by work setting (range 19–100%) and occupation (range 4–100%) were noted in the study. The problem with HBV vaccination in Croatia is that subsequent confirmation of immunization by HBV surface antigen testing is not mandatory. Therefore, it is not known whether a particular HCW is protected from infection until routine hepatitis B titer is performed following occupational exposure to blood. For the same 6-year period, only two HCWs with occupational HBV-infection were reported by the Croatian Institute for Health Protection and Safety at Work [32].

Many countries recommend the vaccination of HCWs against influenza. In recent years, there has been an increase in the vaccination rates among HCWs in some countries, although not in others [33]. The average vaccination rate of HCWs in Croatian hospitals during the initial years of monitoring (2006–2009) was approximately 30% (range 25–36%), which corresponded to the vaccination rates in other countries [5,16]. Educational activities, vaccination campaigns, easy access to free vaccines and the use of formal declination forms have been shown to increase vaccination rates in some countries to as high as 80% [16,33].

In order to increase the HCW awareness concerning the need for immunization, many educational activities are organized by the Ministry of Health, CNIPH and professional societies of the Croatian Medical Association, including information on the real burden of adverse events and the safety of the vaccines. Regarding blood-borne infections, including protection from hepatitis B, national guidelines were published in 2004 [12], and since 2007 a pilot project of the Ministry of Health, Investigation of the Risk of Occupational Exposure to Blood-Borne Infections among Personnel in Croatian Hospitals, has been implemented, which includes educating HCWs through leaflets, posters, lectures, courses and workshops on pre- and post-exposure prophylaxis. For the education of HCWs on immunization against influenza, the CNIPH, in addition to the aforementioned educational activities, has opened a website with information and answers to frequently asked questions. Every year, the CNIPH commemorates Immunization Week, a conference for the professional societies of the Croatian Medical Association [34].

Significant differences in vaccination coverage exist among specialties and employee groups: physicians and medical students are more likely to be vaccinated than nurses, nursing aides and administrative personnel [35]. Our study has shown that according to professions, the highest rate of immunization was among physicians and the lowest among practical nurses. Even housekeepers had a higher rate of immunization. Actually, coverage rates among practical nurses (the largest group of HCWs, accounting for 42–55% of all hospital HCWs) compared to physicians and graduate nurses were 30% and 45% lower in the pre-pandemic and post-pandemic periods, respectively. This results in inadequate vaccination rates among those with the greatest amount of patient contact, potentially providing a basis for group-specific interventions.

A number of studies have addressed behavioral responses to the 2009 influenza pandemic among HCWs. Overall, uncertainty about vaccine side effects, concern about vaccine safety and distrust of the health authorities were the most common reasons stated for non-vaccination [36,37]. Although vaccination against seasonal influenza among HCWs in Croatia was 30% in 2009, the vaccination rate against pandemic influenza in same year was <5%. This can partially be explained by the fact that vaccination started at the peak of the influenza pandemic, although it is undoubtedly partially due to increased concern about the potential side effects of the vaccine and the impact of an anti-vaccination campaign [38].

A systematic review that summarizes the results from 20 publications sampling HCWs from various geographical regions showed that pandemic vaccine coverage was variable (9–92%) across HCW populations [39]. The most important sociodemographic predictor of vaccine uptake was found to be previous seasonal influenza vaccination [39,40]. HCWs were likely to accept the pandemic vaccine if they perceived H1N1 as a serious and severe infection, and considered the pandemic vaccine to be safe and effective in preventing infection to themselves and others (e.g., loved ones, co-workers and patients) [39].

In the first post-pandemic season, 2010–2011, there was a significantly lower seasonal influenza vaccination rate (total decline of 24%) in comparison to pre-pandemic season. This negative trend continued during the following season, 2011–2012 (a decline of 35% in comparison to 2009–2010).

Among the reasons cited by HCWs for why they were vaccinated, self-protection was in the first place, the protection of family members in the second, while the protection of patients was lower on the list of priorities [40]. Since patient welfare is not a sufficiently motivating factor for HCWs to choose influenza vaccination, the introduction of mandatory vaccination is a possible option. The main justification for mandatory vaccination stems from the duty of caregivers to protect their patients [41]. Current guidelines of the Infectious Diseases Society of America state that annual influenza vaccination should be mandatory for HCWs in the interest of safeguarding patients and protecting public health [42].

In the same way that HBV vaccination was introduced into the general population, there have been attempts to introduce mandatory influenza vaccination for the entire population, which resulted in a reduction in influenza-associated mortality and healthcare use [43].

The main limitation of this study is its observational nature, which resulted in some differences in the quality of the reported data, although the numbers of hospitals and HCWs included represent a significant part of the HCWs in Croatia. However, this is the first study that reports influenza and HBV vaccination rates in Croatian hospitals. Future research should include better stratified samples, expanded activities and personnel in community and outpatient healthcare institutions, especially emergency medical services with higher rates of exposure.

## Conclusions

Substantial variation across hospitals and different categories of HCWs was observed in vaccination coverage for both seasonal influenza and HBV. However, HBV vaccination coverage is quite satisfactory compared to seasonal influenza vaccination coverage among healthcare personnel in Croatia. A possible reason for this difference is that HBV vaccination is mandatory. These findings highlight the need for national strategies that optimize vaccination coverage among HCWs in Croatian hospitals, including mandatory vaccination for seasonal influenza.

## Abbreviations

CNIPH: Croatian national institute of public health; HBV: Hepatitis B virus; HCWs: Healthcare workers; NHICAC: National hospital infection control advisory committee.

## Competing interests

The authors declare that they have no competing interests.

## Authors’ contributions

RC, IK, VS and SK developed the research question and protocol, and conducted the analyses. RC, NP and JC drafted the manuscript. NP and JC were involved in the conception and design of the study, acquisition of data, and analysis and interpretation of data. RC and NP performed the statistical analyses. All the authors revised the manuscript and approved the final draft.

## Authors’ information

RC, VS and SK are members of the National Hospital Infection Control Advisory Committee of the Croatian Ministry of Health.

## Pre-publication history

The pre-publication history for this paper can be accessed here:

http://www.biomedcentral.com/1471-2334/13/520/prepub
